# Ecotypic variation for seed dormancy, longevity and germination requirements in wild/weedy *Sorghum bicolor* in Ethiopia: implications for seed mediated transgene dispersal and persistence

**DOI:** 10.1186/2193-1801-2-248

**Published:** 2013-05-30

**Authors:** Asfaw Adugna

**Affiliations:** Melkassa Agricultural Research Center, P.O. Box 1085, Adama, Ethiopia

**Keywords:** Dormancy, Ecotype, Gene flow, Persistence, Seed, Transgenic, Wild sorghum

## Abstract

Seed dispersal is one of the vehicles of gene flow in plants. If a seed carrying transgene(s) is dispersed into the environment, the fate can be determined by its persistence in the soil bank, which can also vary in different ecotypes of a species and the physical environment of the soil including temperature and moisture. This study aimed at investigating ecotypic differences in wild sorghum for dormancy and longevity and their response to varying levels of temperature and moisture for seed germination to aid efforts to predict the potential risk of transgene flow via seeds and persistence in the soil. Wild sorghum seed was collected from different geographical regions in Ethiopia and buried for a maximum of 24 months in the soil. In a separate study, three levels of constant temperature and five levels of osmotic potential (Ψ_s_) were used to investigate variation in wild sorghum ecotypes for seed germination. Viability of buried seeds declined over time, but the rate of decline differed among ecotypes. Better seed longevity was observed at 20 cm soil depth than 10 cm in two wild sorghum ecotypes. Crop seeds depleted within the first six months regardless of the burial depth whereas on the average 1.24% viability was observed in wild sorghum seed after 24 months of burial in the soil. Ecotypic differences were also evident in response to varying temperature and Ψ_s_. Therefore, dispersed seeds carrying crop genes (including transgenes) could persist in the soil for considerable period of time, which may have implications for transgene flow and persistence.

**Electronic supplementary material:**

The online version of this article (doi:10.1186/2193-1801-2-248) contains supplementary material, which is available to authorized users.

## Introduction

One of the central features of seed dormancy is to maintain a reservoir of soil seed bank for future re-establishment following unfavorable conditions (Monaghan 1979). Dormancy is one of the major mechanisms by which weeds in agricultural fields invade and persist (Narwal et al 2008). Sorghum seeds exhibit dormancy at harvest (Gaber et al 1974), but after ripening is known to improve germination in the majority of wild sorghum species by breaking this dormancy (Sastry et al 2006). Moreover, treatment with 0.5% and 1% nitric acid (KNO3) was found to improve germination up to 44% in wild sorghum (Shanmugavalli et al 2007). On the other hand, with no dormancy, sorghum seeds stored in ordinary seed packets under dry and high temperature can remain viable up to 12 years (Karper and Jones 1937). An important aspect frequently overlooked in studies of seed dormancy is the possibility that the seed dormancy strategies differ among ecotypes (Bennett 1999). So far, no study has assessed whether wild/weedy sorghum has been able to evolve differences in their seed dormancy and longevity patterns enabling adaptation to particular environments in its center of origin, yet such knowledge may be useful to understand the weediness potential of different wild sorghum populations and also to design effective management methods. Moreover, environmental factors affecting germination, particularly whether or not exposures to high soil temperatures or soil moisture regimes are required for germination have not been determined. In Ethiopia, the range of environments where wild/weedy sorghum exists is wide. It is, thus, likely that wild sorghum in different environments have adapted differently in their response to different seed germination requirements at that site.

Weed seeds buried in the soil have been concern to agriculturalists (Omami et al. 1999) because soil seed banks constitute a key component of plant population dynamics, community structure and annual life-cycles influencing population size within and between years. Seed banks can serve as pools of genetic material, enabling a range of responses to environmental variability, and can buffer populations against temporarily extreme adverse conditions (Teo-Sherrell et al. 1996). Seed-bank persistence between years also allows plants to average their success over time, by reducing both opportunity and risk (Cohen 1966). Seed characteristics such as longevity and dormancy enable populations to survive for several years (Baker 1974). This property of seeds can vary among ecotypes of a species (Monaghan 1979) and are dependent on the environment because they are highly responsive to environmental conditions and may strongly influence the evolution of post-germination life-history characters beyond their role in reducing risk (Donohue 2002). Soil temperature is known to strongly influence both percentage and time of germination of sorghum seed (e.g., Kanemasu et al. 1975). Moreover, the response of wild sorghum seed to varying osmotic potential and temperature could perhaps be helpful to build hydro-thermal models (e.g., Bradford 2002; Rowse and Finch-Savage 2003) to predicting seed germination.

Reports of studies in different species and ecotypes showed variable results for persistence of seeds in the soil. The duration of loss of viability in buried seeds may range from days in the seeds of the composites (Marks and Nwachuku 1986) to nearly 20 years in American dragonhead, common lambs quarters, Pennsylvania smartweed, and shepherd’s-purse (Conn et al. 2006). In between reports vary with species and depth of burial. Narwal et al. ( 2008) found total loss of viability of ryegrass seeds after 16 months of burial at five and 10 cm depth in different soil and simulated rainfall conditions. Noldin et al. ( 2006) compared the seed longevity of several ecotypes of wild rice from four US states and found <1% viable seeds in three ecotypes at 5 cm depth, but nine ecotypes had similar proportion of viable seeds at a depth of 25 cm after two years of burial at two locations in Texas, but within each depth there was minor variation among ecotypes. Burnside et al. ( 1977) also reported that Nebraska farmers noticed wild sorghum (shatter cane) plants 8 years after the field had been planted with Lucerne (*Medicago sativa* L.).

Although both pollen and seed are the two major vehicles of gene flow, they are basically related to each other as volunteer seeds can grow and contribute to pollen mediated gene flow (Nielson et al. 2009). Spatial seed dispersal by natural means is often considered to be low because most crops have lost their ability for independent seed dispersal (De Wet and Harlan 1975). However, seed dispersal can be aided by animals, water, wind, humans via agricultural implements, seed marketing, application of cattle manure, etc. This dispersal can be an important step in the dynamics of crop-wild gene exchange (Jenczewski et al. 2003). The initial level of seed dormancy, longevity and the response of seed germination with respect to environmental flux can influence the potential temporal dispersal of transgenes (Linder and Schmitt 1994). If gene dispersal is known to have occurred via seed, the next step is to study the persistence and further establishment (Jenczewski et al. 2003). The role of soil seed bank dynamics in seed mediated gene flow is quite substantial (Nielson et al. 2009) as soil seed banks are the reservoirs of genetic material upon which natural selection can act (Simpson et al. 1989). Therefore, studying this dynamics is crucial for risk assessment of transgene flow.

Dormancy distributes seed germination in time and space and promotes survival (Li and Foley 1997). Thus, the study of seed dormancy and longevity is useful because it is a fundamental element for determining seed bank dynamics (Van Mourik et al. 2005). As a result, characterization of the germination ecology of ecotypes would help to indicate their potential to become noxious weeds in different systems of cultivation (Buckley et al. 2004). Therefore, knowledge of seed longevity and dormancy can help weed management (Martinez-Ghersa et al. 1997). On the other hand, studies related to ecotypic variation in wild plants are considered to be essential for conservation of biodiversity (Crandall et al. 2000) in order to avoid extinction associated with environmental change (Fraser and Fraser 2000). In addition, populations of the same species that grow in different locations may show local adaptation to site conditions. Therefore, the objectives of the present study were 1) to assess ecotypic variation for dormancy and longevity of buried wild/weedy sorghum seeds, 2) to assess ecotypic variation in the response of wild sorghum seeds collected from different geographical regions in Ethiopia to different temperature and moisture regimes, and 3) to predict the potential risk of seed mediated transgene dispersal and persistence to aid efforts to conserve wild sorghum genetic resources.

## Materials and methods

### Effect of burial time and depth on seed longevity of different ecotypes of wild sorghum

This study consisted of two experiments:

#### Experiment-1

Seed samples were collected in 2008 from five populations of wild and weedy sorghum (*S. bicolor* L.) subsp. verticilliflorum and subsp. drummondii representing five geographical regions of collection (Ghibe river valley, Hararghe, Metekel, Tigray, and Wello) in Ethiopia (Table 1). Each population (hereafter referred to as ecotype) was represented by 5 individual plants. It was a two factor factorial experiment in randomized complete block design (RCBD) with four replications. The factors were 25 genotypes and 4 exhumation dates. A total of 400 bags were originally buried during the dry season on 14–15 March 2009 at Melkassa Agricultural Research Center. Every six months (on 16 September 2009, 26 February 2010, 5 October 2010, and 16 March 2011), twenty-five genotypes from 4 replications (total 100 bags) were exhumed for viability test.

**Table 1:** **Geographical characteristics of the wild sorghum collection sites**

Geographical region	Name of location	Long. (N)	Lat. (E)	Alt. (masl)
Ghibe	Bhede-Dhero	8° 10’	37° 33’	1686
Hararghe	Kara Kurkura/ Gerbi	8° 52’	40° 42’	1709
Metekel	Mandura	11° 05’	36° 25’	1404
Tigray	Hagereselam-Idris	14° 05’	36° 58’	820
Wello	Aradom	12° 06’	39° 38’	1460

#### Experiment-2

In this part of the study, seed from three to four plants from each of the two wild sorghum populations collected from Mandura and Ghibe in November, 2009 was bulked. Moreover, one cultivated sorghum variety, WSV 387 was included. The treatments were arranged in a factorial experiment in RCBD with three replications. The three factors were two burial depths (10 cm and 20 cm), three populations (two wild and a cultivar) and three exhumation dates (6, 12, and 18 months after burial). Therefore, 54 bags were buried on 26 April 2010 and 18 bags were exhumed every six months (on 25 September 2010, 16 March 2011, and 3 November 2011) for viability test.

### Seed burial

For both experiments, double layered permeable nylon mesh bags of size 15 cm × 35 cm were prepared for seed burial to create the natural soil environment. In many of the earlier studies (e.g., Omami et al. 1999; Narwal et al. 2008) a large number of seeds mixed with soils were placed in small bags, usually 10 cm × 10 cm. However, high seed density in the mesh bags may influence seed mortality because of elevation of pathogen levels as it is higher than the natural density in the soil bank (Van Mourik, et al. 2005). In this experiment a more ideal proportion of soil to seed mixture was used as suggested by Teo-Sherrell et al. ( 1996). Accordingly, 50 seeds from the intended samples were mixed with 500 g of sieved soil in the mesh bag. The soil was collected at Melkassa Agricultural Research Center where the species was not grown for more than three years. Paper labels sealed in polythene bags were placed in each mesh bag for identification of samples during exhumation. In order to prevent loss of seeds from the bags and to avoid entrance of soils and other species weed seeds, the bags were tightly fastened and buried in horizontal position. The bags were buried with their colored strings extended to the outside of the soil for easy detection of the bags during exhumation as suggested by Van ( 2005). They were buried 50 cm apart from one another in a grid. The field was previously used for conducting other crop experiments and has been extensively disturbed. However, during the duration of the experiment it was kept undisturbed (no cultivation).

### Viability test

After each exhume, the seeds were sorted from the soil with 2 mm sieve and graded based on their physical appearance and strength. During the first exhume in both experiments, the seeds that germinated in the field were found with their intact dead plumule and radicle tissues. Moreover, some seeds died in the field as evidenced by their brown endosperm and the easiness with which they crushed when minimum force was applied to them. However, starting from the second exhume it was difficult to identify those seeds, which germinated in the field as the dried tissues decomposed. The stiff seeds were washed and kept in germination boxes and soaked in 2% Clorox for 5 minutes in order to sterilize them and rinsed twice with distilled water. The seeds were then placed in new 11 cm × 11 cm × 3.5 cm germination boxes on blotters wetted with distilled water. The germination boxes were placed in incubator adjusted at 30°C temperature, which was selected based on calibration. The germinated seeds were counted and removed starting from the third day for two weeks. Later, the non germinated seeds were tested for viability by dissecting and soaking them in 1% Tetrazolium chloride (2,3,5-triphenyltetrazolium chloride) solution for 6–8 hours at 35°C in an incubator. Seeds that were fully or >2/3 stained (red) were considered viable. Therefore, the whole data in each set was classified in to three groups: germinated in lab (germinable), dormant and dead. The dormant seeds group included those seeds which didn’t germinate during lab germination test, but were found viable using TTC test. The dead group included seeds those germinated and/or decayed in the field and those found intact but confirmed dead after TTC test. All of the proportions (percentage) of the viability measures were estimated based on the original 50 seeds. Data on mean monthly soil temperature at 10 cm and 20 cm depth and rainfall measurements were recorded at a meteorological weather station of the research center located about 200 meters from the experimental plot.

### Response of wild sorghum ecotypes to differential osmotic potential and temperature regimes

This study was carried out in September, 2011 at Melkassa Agricultural Research Center. Polyethylene glycol (PEG 6000) was received from Department of Evolution, Ecology and Organismal Biology, Ohio State University, USA to create osmotic potential. It was a three factor factorial experiment in a randomized complete block design (RCBD) with four replications. The factors were osmotic potential (Ψ_s_) with five levels (0 MPa (control), −0.3 MPa, −0.6 MPa, −0.9 MPa, and −1.2 MPa), three constant temperature levels (15°C, 23°C and 30°C) and three wild sorghum ecotypes (collected from Kara Kurkura/ Gerbi, Mandura, and Aradom). The above levels of Ψ_s_ were produced by mixing 0 g (distilled water as control), 135.6 g, 204.4 g, 257.6 g, and 302.5 g at 15°C; similarly, 0 g, 148.1 g, 219.5 g, 274.7 g, 321.2 g at 23°C; and 0 g, 160.5 g, 234.7 g, 291.6 g, and 339.7 g of polyethylene glycol (PEG 6000) at 30°C in a liter of distilled water following the formula of Michel and Kaufmann ( 1973) as follows:

Where, Ψs = osmotic potential in Mega Pascal (MPa), C = PEG concentration, T = Temperature. The minus sign indicates that dissolved PEG reduces the water potential of a solution relative to the reference state of pure water (Tazi and Zeiger 2003).

The wild sorghum genotypes (ecotypes) used in the study were grown at Melkassa Agricultural Research Center during the 2010 main rainy season under the same management and harvested in November of the same year. The seeds were kept in the laboratory at room temperature (25 ± 3°C) until the beginning of the experiment. The genotypes were selected based on their representation to three different agro ecologies and by their high rate of germination (≥88%). In order to avoid mechanical seed dormancy, the glumes were removed from the seeds before the experiment using fine sandpaper. Twenty-five seeds from each of the three wild sorghum genotypes in each replication were placed in 115 mm Petri dishes on top of Whatman® number one filter paper. To avoid decay of seeds by fungi, seeds were treated with Apron Star®. Five milliliter of each PEG solution was applied to each Petri dish and the Petri dishes were sealed with a two inch wide Para film® to avoid evaporation. The Petri dishes were randomly placed in each of the two upper compartments of a WTC binder 78532 incubator (Tuttlingen/ Germany) in the dark. In order to avoid breakage of the Para film, one liter of distilled water was kept in a 5 liter beaker at the bottom compartment of the incubator to raise the humidity.

### Data recording and statistical analysis

For all experiments, germination data were recorded every day starting from the third day until day 15. A seed was considered germinated if the radicle was at least 1 mm long. All germinated seeds were removed after counting. The germination and TTC viability data were converted into percentages of the total seeds, 50 in each bag, for the seed burial experiment and 25 for the experiment involving temperature and osmotic potential. Total viability was computed as the percent of seeds germinated in the lab plus percent of dormant seeds (TTC viable). General Linear Model (GLM) ANOVA with a Poisson distribution was computed on the arcsine transformed data using MINITAB software release 14. Replication was considered as random effect and genotype, depth, temperature, osmotic potential, and duration of burial were regarded as fixed effects. Analysis was done separately for each of the three data sets, experiment-1 and experiment-2 of the seed burial study, and for the experiment involving temperature and osmotic potential. For all experiments, survival curves were plotted. Because most of the genotypes had zero viability at the 24^th^ month, the data in set-1 recorded at the final exhumation date was excluded from GLM ANOVA. Because the viability of buried seeds in soils follow exponential decline (Conn et al. 2006; Nielson et al. 2009), the rate of decline was computed from the exponential decay function as:

Where, P_(t)_ = the number of seeds still viable after time t, P_0_ = the initial number of seeds buried, r = the decay rate, and t = the time (duration) of burial (in years) in the soil. Moreover, the time taken for 50% of the seeds to lose their viability (half life or t_0.5_) was calculated using Half Life Calculator, a web based program available at http://www.calculator.net/half-life-calculator.html (accessed on 16 April 2012) from the above formula:

## Results

### Fate of wild sorghum seed after 18 and 24 months of burial in the soil

General Linear Model (GLM) ANOVA showed significant difference (p < 0.05) for the Genotype × Duration of burial interaction (Additional file 1), which indicates that genotypes (ecotypes) collected from different geographical regions showed differential response on seed viability (longevity) to the different periods of burial. Some of them showed drastic decline in viability or total germination in just a few months, others showed relatively slower, but ultimately, most of the seeds were depleted within the 24 months of burial period.

**Figure 1:**
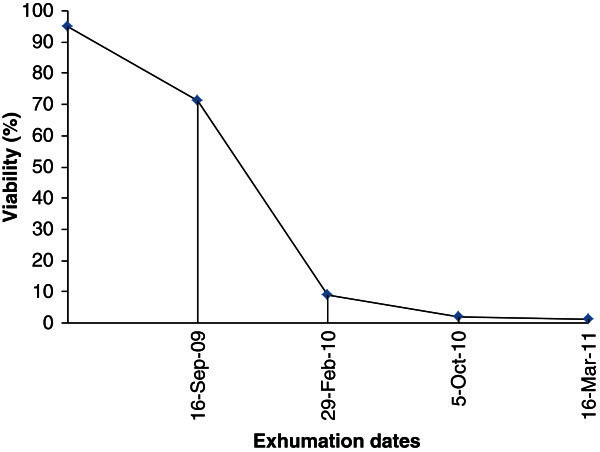
**Survival curve based on pooled average seed viability data of the five wild sorghum ecotypes after 24 months of burial in the soil.**

The data of all populations in experiment-1 pooled together, 71% (SD = 4.19) of all the seeds were viable of which 49.7% (SD = 20.44) were germinated in the laboratory, 21% (SD = 16.28) were dormant and 24.14% (SD = 4.40) were germinated and/or dead in the field during the first 6 months of burial (Table 2). The remaining 4.8% (SD = 3.56) of the seeds were found intact but were confirmed dead using 1% TTC test. Hence, a large proportion of the seeds were found to be viable after 6 months of burial in the soil. After 12 months of burial, however, the viability sharply declined to 9.06% (SD = 7.78) of which 2.32% (SD = 1.39) were dormant. After 18 months of burial, only 1.82% (SD = 1.95) of the seeds remained viable of which 1.34% (SD = 1.31) were still dormant. Furthermore, after 24 months of burial, 1.24% (SD = 2.0) remained viable, of which 0.74% (SD = 1.05) were still dormant. Figure 1 shows the survival curve of wild sorghum seeds during the 24 months period of burial in the soil (Experiment-1). On the basis of the total viability of seeds in the entire population, ecotypic variation in the wild sorghum populations was not evident during the first six months of burial, but there were differences among ecotypes for dormancy (the proportion of seeds that confirmed viable by TTC test, but did not germinate). The highest dormancy was recorded by Ghibe (39.1%, SD = 16.28) and Pawe (38.5%, SD = 16.28) populations, but the lowest (4.2%) was by Hararghe population. After a year of burial, Ghibe population showed better total viability than the rest of the populations included in the experiment followed by Pawe. During the third exhume, Pawe and Ghibe populations still showed better total viability than the remaining populations. After 24 months of burial, Ghibe populations had an average viability of 4.8% (SD = 2.0), 2.6% (SD = 1.05) of which were still dormant. The other populations had <1% viability.

**Table 2:** **Survival of buried seeds** (**experiment**-**2**) **from five populations** (**ecotypes**) **of wild sorghum after 6**, **12**, **18**, **and 24 months**, **based on pooled data from 20 mesh bags per population**

Population	Mean percent of viable seeds	Percent germinated seeds in lab (non-dormant)	Percent TTC viable seeds (dormant)	Percent intact but dead seeds (TTC non-viable)	Percent germinated/decomposed in field
Excavated after 6 months of burial					
Ghibe	67.1	28	39.1	10.2	22.7
Hararghe	75.2	71	4.2	2.5	22.3
Pawe	66	27.5	38.5	2.5	31.5
Tigray	73.6	60.2	13.4	2.1	24.3
Wello	73.4	61.7	11.7	6.7	19.9
Mean	71.06	49.7	21.38	4.8	24.14
SD	4.19	20.44	16.28	3.56	4.40
Exhumed after 12 months of burial					
Ghibe	22.5	17.8	4.7	8.1	69.4
Hararghe	4.8	2.6	2.2	3.4	91.8
Pawe	9.2	7.7	1.5	5.4	85.4
Tigray	4.3	3.1	1.2	0.5	95.2
Wello	4.5	2.5	2.0	1.0	94.5
Mean	9.06	6.74	2.32	3.68	87.26
SD	7.78	6.55	1.39	3.16	10.71
Exhumed after 18 months of burial					
Ghibe	3.4	1	2.4	3	96.6
Hararghe	0	0	0	0	100
Pawe	4.3	1.4	2.9	3.2	95.7
Tigray	1.3	0	1.3	1.5	98.7
Wello	0.1	0	0.1	1	99.9
Mean	1.82	0.48	1.34	1.74	98.18
SD	1.95	0.67	1.31	1.36	1.95
Exhumed after 24 months of burial					
Ghibe	4.8	2.2	2.6	12.8	82.4
Hararghe	0.2	0	0.2	0.9	98.9
Pawe	0.7	0.2	0.5	6.1	93.2
Tigray	0.2	0.1	0.1	0.7	99.1
Wello	0.3	0	0.3	0.5	99.2
Mean	1.24	0.5	0.74	4.2	94.56
SD	2	0.95	1.05	5.35	7.26

**Figure 2:**
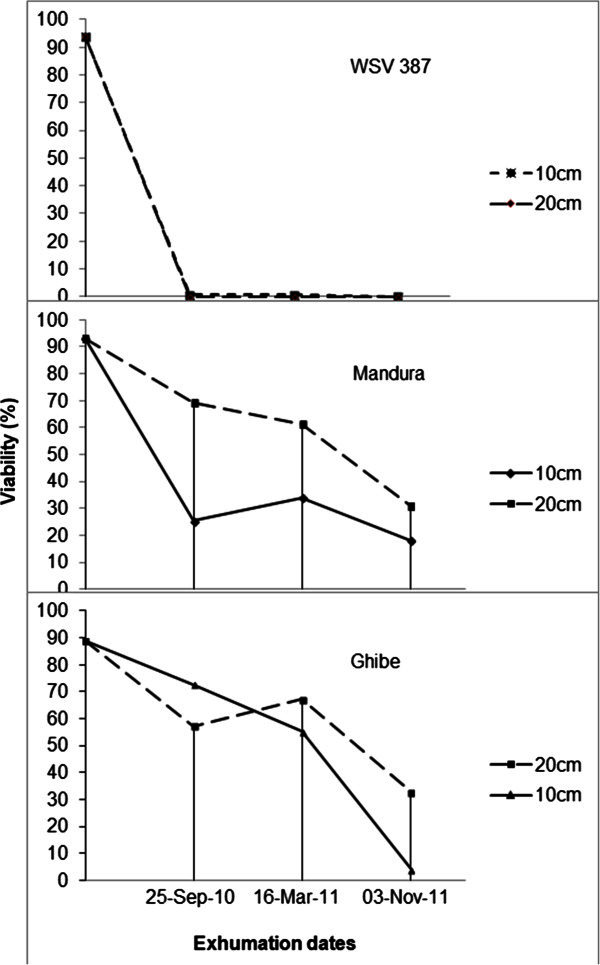
**Effect of burial time as measured by exhumation period and depth on viability of wild sorghum seed collected from Ghibe and Mandura and cultivated sorghum variety WSV 387.**

The second-order interaction (Genotype × Depth × Duration of burial) in experiment-2 was highly significant showing that different populations/ecotypes had different viability after some period of burial at different burial depths. Differences for viability associated with dormancy were highly significant only for genotype factor (Additional file 2). In general, crop seeds totally germinated and/or died within the first 6 months regardless of the depth of burial (Figure 2). However, wild sorghum retained their viability up to 31% and 33% in Ghibe and Pawe populations, respectively after 18 months of burial at 20 cm soil depth (also Table 3). At a depth of 10 cm the rate of decline in viability was sharp in Ghibe population, but Pawe population showed an increased viability after a year of burial. On the contrary, this kind of increased viability after a year at 20 cm depth was observed in Ghibe population (Table 3).

**Table 3:** **Percent of viable seeds**, **the rate of decline and the time it takes for 50% of wild and cultivated sorghum seeds to lose viability** (**half**-**life**) **after 0**.**5**, **1 and 1**.**5 years of burial in the soil**

Population	Duration of burial, t (years)	10 cm depth			20 cm depth		
		Average viable seeds (%)	Rate of decline (r)	t_(0.5)_	Average viable seeds (%)	Rate of decline (r)	t_(0.5)_
Ghibe	0.5	72.7	0.64	1.085	57.3	1.11	0.623
	1	55.3	0.59	1.171	67.3	0.40	1.753
	1.5	4.0	2.15	0.323	32.7	0.75	0.929
Pawe	0.5	25.3	2.75	0.252	69.3	0.73	0.946
	1	34.0	1.08	0.643	61.3	0.49	1.418
	1.5	18.0	1.14	0.606	30.7	0.79	0.880
WSV 387	0.5	0.7	10.02	0.069	0.0	0.60	0
	1	0.7	5.01	0.138	0.0	0.60	0
	1.5	0.0	0.60	0.087	0.0	0.60	0

### Response of wild sorghum ecotypes for varying moisture and temperature regimes

**Figure 3:**
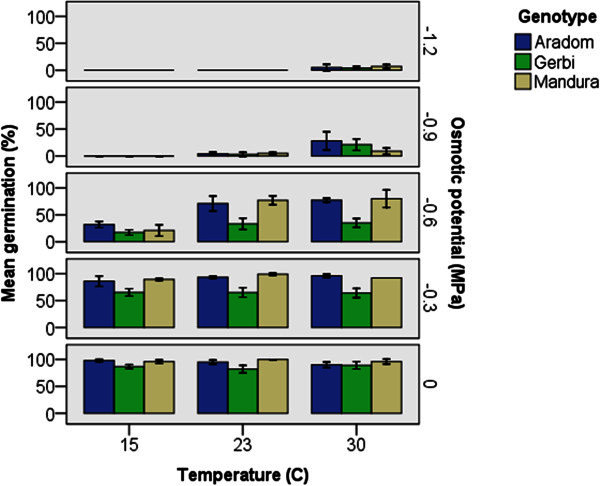
**Response of wild sorghum ecotypes for varying levels of osmotic potential and temperature.** Vertical bars are ± SE.

General Linear Model ANOVA showed that differences among all factors and their interactions were significant (p < 0.01) (Additional file 3). The three genotypes revealed differential response to the varying levels of temperature and moisture regimes for seed germination. At all temperature levels, germination was greater or equal to 80% in all the genotypes under no osmotic potential (control) condition. In each genotype there was a trend of increase in germination with an increase in temperature in each level of osmotic potential. Accordingly, at the lowest temperature (15°C), all of the three genotypes showed no germination at Ψ_s_ lower than −0.6 MPa (Figure 3). At 23°C all of them showed germination at Ψ_s_ of −0.9 MPa or higher, and at 30°C, all of them showed germination at all levels of osmotic potential. In general, there was a trend that decrease in osmotic potential (increase in moisture stress) decreased seed germination in all genotypes, but the extent was different with temperature and genotype. At the highest temperature (30°C) the Wello population from Aradom showed the highest germination when there was mild osmotic potential (Ψs = −0.3 MPa) (Figure 3). This germination was comparable to the highest germination in the control treatment levels at the other two temperature levels for the same population. Similarly, at Ψs = 0.6 MPa, this population showed better germination than the other two populations.

## Discussion

### Effect of burial time and depth on seed germination of wild and cultivated sorghum genotypes

The result of the present study on the fate of wild sorghum seed after 18 and 24 months of burial was in agreement with the results of Jacques et al. ( 1974) who did similar study in Kansas. They found that only few seeds of cultivated sorghum remained viable after four months of burial regardless of the burial depth, but some shatter cane seeds were able to survive for three years mostly from deeper burial depths (30 cm to 38 cm). The result was also intermediate between the results of Burnside et al. ( 1996) and Teo-Sherrell and Mortensen ( 2000). Burnside et al. ( 1996) found little viability in weedy sorghum seed after five years of burial, but they also observed 1% germinated after 17 years of burial in eastern Nebraska. Teo-Sherrell and Mortensen ( 2000) reported more than 80% mortality in weedy sorghum seed within three months after burial (between November and March) in Nebraska because of winter cold. However, such kind of winter environment of the temperate regions does not exist in most sorghum growing regions of Sub-Sahara Africa including Ethiopia. The observation that an increase in depth of burial increases longevity was in agreement with the previous reports in sorghum (Jacques et al. 1974), crimson and red clovers (Rampton and Ching 1970), in jointed goat grass (Donald and Zimdahl 1987), in wild oat (Miller and Nalewaja 1990), and in *Amaranthus retroflexus* seeds (Omami et al. 1999), to mention a few. This may be due mainly to the unavailability of suitable conditions for germination such as a decrease in oxygen content with increase in depth. In addition, seeds near the surface of the soil are more liable to natural predators and greater fluctuation in environmental conditions, which may contribute to faster rate of decay (Chantre et al. 2008).

The fact that differences for viability associated with dormancy were highly significant only for genotype factor may indicate that dormancy is mainly a characteristic of the plant (genotype). It could be largely influenced by the physiological and genetic makeup within the seed. Dormancy is known to be controlled by many genes (Li and Foley 1997). Similarly, Dunbabin and Cocks ( 1999) reported large differences in the seed dormancy pattern of two ecotypes of cape weed (*Arctotheca calendula*) in Western Australia. The two wild sorghum populations showed increased viability at the two depths of burial differently after a year, but both of them showed decline in viability thereafter (Table 3). This may be due to the dormancy-non-dormancy cycle as observed in other plant species (e.g., Baskin and Baskin 1988). In general, crop seeds totally germinated or died within the first 6 months regardless of the depth of burial. The difference in longevity between the crop and the wild seeds in the present study could be attributed to different reasons. First, it may be due to conditions of the seed that are inherent. Secondly, it may be related to differences in their initial level of dormancy. The wild sorghum seeds from Ghibe and from Pawe had 35% and 45% dormancy, respectively before burial whereas the cultivated sorghum had near complete germination (94%) (Data not shown). This much dormancy in the wild was observed after five months of storage in the laboratory at room temperature (after ripening) as seed dormancy is related to longevity (e.g., Jurado and Flores 2005). The third reason could be due to mechanical dormancy as a result of seed covering by glumes. This behavior of wild sorghum seeds was also observed in earlier studies and degluming was found to improve germination (Sastry et al. 2006). Similar observation was also reported in wild rice (Gu et al. 2003).

Soil temperature at Melkassa seemed to have less effect to bring about variation in dormancy among the different exhumation periods as the trend was more or less similar during the duration of burial (Additional file 4). However, the rainfall distribution might have considerable effect (Additional file 5). After burial of bags for experiment-2 in April 2010, there was high rainfall; even the highest rainfall during the duration of burial was in July-August of the same year, which might have contributed to the depletion of crop seeds by germination within the first 6 months of burial.

### Response of wild sorghum ecotypes for varying osmotic potential and temperature regimes

Populations of wild sorghum collected from different geographical regions (ecotypes) were found to show some level of variation in their germination requirements. Accordingly, with no osmotic stress, Aradom population showed the highest mean germination (98%) at 15°C whereas at 23°C and 30°C, Mandura population showed the highest mean germination (100% and 96%, respectively) (Figure 3). The germination of Gerbi population was the lowest at all temperature levels. Despite their differences, the seeds of all ecotypes did not germinate at 15°C when Ψs was < −0.6 MPa and at 23°C when Ψs was < −0.9 MPa. Smith and Hoveland ( 1986) also found 44% reduction in germination of cultivated sorghum seed when Ψ_s_ decreased from 0 to −1.0 MPa. In agreement with this study, Smith et al. ( 1989) observed no germination at 15°C and at Ψ_s_ of −1.2 MPa in cultivated sorghum and in pearl millet. Similarly, Lippai et al. ( 1996) studied the response of Horehound seed at different temperature and osmotic potential and found increased germination for each degree increase in temperature but decreased with decreased Ψ_s_ and ceased at −1.5 MPa. The superior germination observed in Aradom population over the other two populations at the highest temperature (30°C) with mild moisture stress (Ψs = −0.6 MPa) might be because this wild population is adapted to the dry lowlands characterized by high temperature and drought stress conditions. The observed better germination in Aradom population at −0.3 MPa of Ψ_s_ than the control at 30°C may indicate that some level of moisture stress is helpful to enhance seed germination for wild sorghum that are adapted to the dry lowlands. Perhaps this is among the reasons why wild sorghum is predominantly distributed in the lower altitude, moisture stressed areas of Ethiopia than in the highlands (Adugna and Bekele 2013a).

### Implications for seed mediated transgene dispersal and persistence

The authors have been trying to follow various modalities to investigate the risk of crop-wild gene flow and beyond (Adugna and Bekele 2013a 2013b; Adugna et al. 2013a 2013b). The present study was intended to estimate the length of time the wild and crop sorghum seeds can persist in the soil and their germination ecology to give inference of probable survival and persistence of transgenes. It was found that sorghum crop seeds depleted in just a few months, but wild sorghum seeds survived for considerable period of time in the soil bank perhaps because crops might have lost their seed longevity and persistence mechanisms with domestication and breeding. This may also mean that when there is no human interference, crop genes can only escape to the environment and survive via the seeds of their wild relatives as they can freely interbreed. As the resulting hybrids do not show reduced fitness (Adugna and Bekele 2013b), the escaped genes can spread into the environment with no barrier. Therefore, crop genes (including transgenes) have high probability of persistence in the wild once pollen mediated gene flow has occurred between the two and provided the germination requirements are not fulfilled, which calls for effective management of wild seed movement. Moreover, because wild sorghum could serve as reservoirs of valuable genes for future crop improvement, it is important to conserve the observed ecotypic diversity.

## Electronic supplementary material

Additional file 1: **GLM ANOVA of the wild sorghum ecotypes (genotypes) collected from five regions for their longevity.** (XLSX 9 KB)

Additional file 2: **Mean squares from GLM ANOVA showing the effect of burial time and depth on longevity of two wild sorghum ecotypes and a cultivar seed for total viable and dormant (TTC stained) seeds.** (XLSX 9 KB)

Additional file 3: **Mean squares from GLM ANOVA showing the effect of the individual and interaction effects of temperature and osmotic potential on seed germination of three sorghum ecotypes.** (XLSX 9 KB)

Additional file 4: **Monthly average soil temperature at Melkassa for the duration of the seed burial experiment.** (PNG 43 KB)

Additional file 5: **Monthly rainfall distribution at Melkassa for the duration of the seed burial study.** (PNG 51 KB)
