# Supplementary figures and images for: Ecotypic variation for seed dormancy, longevity and germination requirements in wild/weedy Sorghum bicolor in Ethiopia: implications for seed mediated transgene dispersal and persistence
Source: Springerplus. 2013 May 30;2:248. doi: 10.1186/2193-1801-2-248 (PMC3682103; doi:10.1186/2193-1801-2-248)

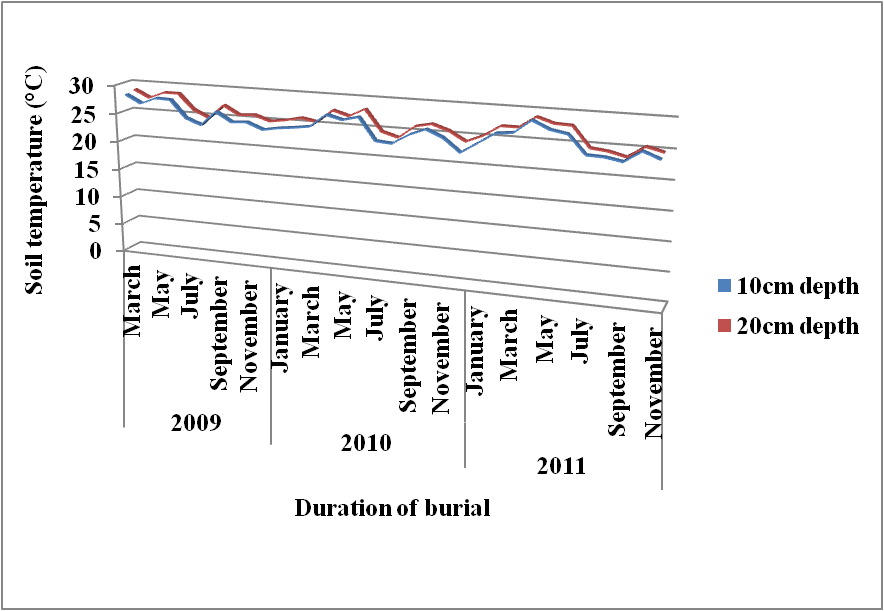

Supplement: Supplementary file 4 — Additional file 4: Monthly average soil temperature at Melkassa for the duration of the seed burial experiment. (PNG 43 KB) [file 40064_2013_316_MOESM4_ESM.png]

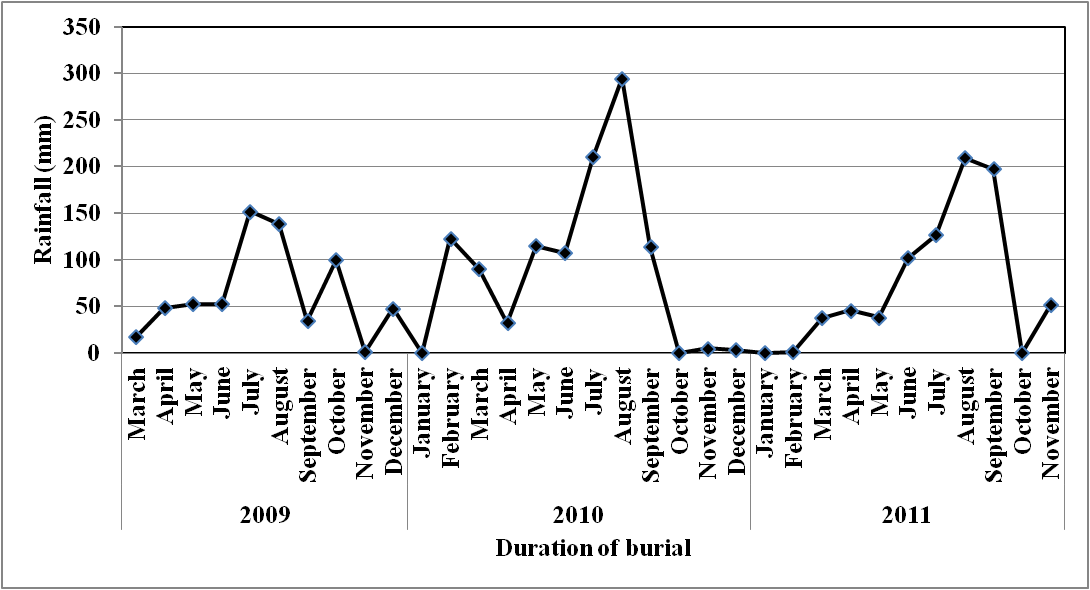

Supplement: Supplementary file 5 — Additional file 5: Monthly rainfall distribution at Melkassa for the duration of the seed burial study. (PNG 51 KB) [file 40064_2013_316_MOESM5_ESM.png]
